# Cyclosporiasis in travellers returning to the United Kingdom from Mexico in summer 2017: lessons from the recent past to inform the future

**DOI:** 10.2807/1560-7917.ES.2017.22.32.30592

**Published:** 2017-08-10

**Authors:** Diogo F P Marques, Claire L Alexander, Rachel M Chalmers, Peter Chiodini, Richard Elson, Joanne Freedman, Gauri Godbole, Gillian Hawkins, Janice Lo, Guy Robinson, Katherine Russell, Alison Smith-Palmer, Hilary Kirkbride

**Affiliations:** 1 Health Protection Scotland (HPS), Glasgow, United Kingdom; 2 European Programme for Intervention Epidemiology Training (EPIET), European Centre for Disease Prevention and Control (ECDC), Stockholm, Sweden; 3 Scottish Parasite Diagnostic and Reference Laboratory (SPDRL), Glasgow, United Kingdom; 4 Cryptosporidium Reference Unit, Public Health Wales, Swansea, United Kingdom; 5 Hospital of Tropical Diseases, London, United Kingdom; 6 Public Health England (PHE), London, United Kingdom

**Keywords:** Cyclospora, cyclosporiasis, gastrointestinal disease, travel, outbreaks, parasitic diseases

## Abstract

During the summers of 2015 and 2016, the United Kingdom experienced large outbreaks of cyclosporiasis in travellers returning from Mexico. As the source of the outbreaks was not identified, there is the potential for a similar outbreak to occur in 2017; indeed 78 cases had already been reported as at 27 July 2017. Early communication and international collaboration is essential to provide a better understanding of the source and extent of this recurring situation.

Over the summers of 2015 and 2016, the United Kingdom (UK) reported outbreaks of the intestinal disease cyclosporiasis in travellers returning from Mexico, mainly from the Riviera Maya and Cancun regions [1,2]. As the source of the outbreaks was not identified, there is the potential for a similar outbreak to re-occur. As at 27 July, 78 cases had already been reported in the UK in 2017, of which 37 (47%) had travelled to Mexico, 20 were awaiting travel history, 14 had travelled to nine other overseas destinations and seven reported no overseas travel [3]. We describe and discuss actions taken in advance of an anticipated outbreak during the summer of 2017.

## Previous cyclosporiasis outbreaks among UK travellers returning from Mexico

In 2015, public health authorities in the UK and Canada reported an outbreak of cyclosporiasis in travellers returning from Mexico; 79 cases were reported in the UK and 97 in Canada [1]. Cases from both countries reported staying at various hotels in the Riviera Maya and Cancun region during the incubation period. Geographical and temporal associations suggested that the outbreak was related to a consumed product distributed throughout the region rather than contamination or hygiene deficiencies in individual hotels [1].

On 29 June 2016, Health Protection Scotland (HPS) was informed of cyclosporiasis among travellers returning from Mexico. Subsequently, cases were also detected among residents of England, Wales, Jersey and Isle of Man. Individuals were considered a probable case of cyclosporiasis if they had a sample date between 1 June and 28 October 2016 and travelled to Mexico in the previous 14 days, and if oocysts were identified in stool specimens by a diagnostic laboratory. Confirmed cases were probable cases confirmed microscopically by a national reference laboratory. Between 1 June and 28 October 2016, a total of 440 cases were reported in the UK, of which 289 (66%) were confirmed. 

Food and travel histories were collected, and 359 (82%) cases reported recent travel history to Mexico, four had travelled to four different countries, two reported no overseas travel and travel history was unavailable for the other 75 cases. The epidemiological investigations demonstrated that the majority of cases that travelled to Mexico had stayed in the Riviera Maya and Cancun regions (n = 231; 64%), often in all-inclusive hotels. Despite extensive descriptive epidemiological investigations in the UK, a formal analytical epidemiological study was not conducted and no specific vehicle was identified. 

In view of the geographical distribution of cases across many hotels, the source of infection was likely to have been a contaminated food or drink item supplied to multiple hotels. In 2015 and 2016, cases were confined to the UK summer seasons (June to September), even though Mexico is a year round destination for UK travellers [4], which implies that the source of infection could be seasonal. Throughout the incident response, Public Health England (PHE) as the UK National Focal Point for the International Health Regulations (IHR) provided regular updates to the authorities in Mexico to help inform local investigations.

## Actions taken by the UK Incident Management Team in June and July 2017

The UK incident management team (IMT) met in May 2017 in advance of an anticipated increase in cyclosporiasis cases to coordinate the UK response and focus on early communications and awareness raising.

With the aim to influence travellers’ behaviours abroad, pre-travel advice was provided through online news stories, and an infographic leaflet was promoted via the National Travel Health Network and Centre (NaTHNaC; http://nathnac.net/), TRAVAX (http://www.travax.nhs.uk/) and Fitfortravel (http://www.fitfortravel.nhs.uk) websites. Contact was made with ABTA, the Travel Association, to raise awareness before the peak holiday period. ABTA in turn shared the information with their members. *Cyclospora* advice was also included as part of the Scottish Government letter to primary care about travel advice before the 2017 summer holiday period.

To improve case ascertainment, health protection teams (HPTs) in the UK were informed of the potential for another increase of cyclosporiasis cases in the summer of 2017 among returning travellers. HPTs were also requested to complete questionnaires with new cases to identify a hypothesis for testing and to inform control measures.

To improve laboratory testing, reminders were sent to diagnostic laboratories to consider testing for *Cyclospora* in cases where individuals had compatible symptoms and travel history. Discussions were also held among UK parasitology reference laboratories to share their confirmatory methodologies (fluorescence and stained microscopy and/or PCR), to review the usefulness of quantitative PCR for monitoring assay development and performance, and consider methods for genotyping and progression of whole genome sequencing.

As cyclosporiasis is likely to occur in travellers to Mexico from countries other than the UK, the World Health Organization (WHO) was informed and alerts were sent on behalf of the UK IMT to European Union (EU) countries via the European Early Warning and Response System (EWRS) and the European Centre for Disease Prevention and Control (ECDC) Epidemic Intelligence Information System (EPIS). A recent ECDC rapid risk assessment has also been published [5]. These alerts summarised previous outbreaks and proposed that laboratories in other EU Member States consider *Cyclospora* as a possible cause of gastrointestinal infection in travellers returning from endemic or outbreaks areas. 

To facilitate cross-country collaboration, communications were initiated with the Mexican health authorities and the results of epidemiological investigations from the 2016 outbreak were shared. 

If non-travel-associated cases are identified in the UK, these will undergo a detailed exposure investigation to identity potential sources as they may be a consequence of imported contaminated foods. 

## Discussion

Cyclosporiasis is a treatable intestinal disease caused by the protozoan *Cyclospora*. It is transmitted through contaminated food, water or beverages and has an average incubation period of 7 days after ingestion of sporulated oocysts. Since the oocysts take approximately 10 days to mature before they are infective, direct person-to-person transmission is unlikely. Patients present with diarrhoea, abdominal pain and fatigue [6,7]. Symptoms are usually self-limiting but may persist for several weeks and can be particularly severe in those who are immunocompromised. Cyclosporiasis is endemic in many tropical and subtropical regions including Mexico [8-10] and previous outbreaks have been associated with contaminated fresh produce such as basil [11], cilantro [12], lettuce [13], snow peas [14], raspberries [15] and drinking water [16].

The UK investigations in 2015 and 2016 identified several issues, which the UK IMT have tried to address before the summer of 2017. Firstly, *Cyclospora* is not routinely tested for in primary diagnostic laboratories in the UK and requires additional microscopical procedures to identify the parasite. Testing is usually restricted to diarrhoea cases with travel to endemic (tropical) countries, patients with a specific request form from a clinician or when clinical details are suggestive of *Cyclospora*. Maintaining competencies in identifying the parasite can be difficult when laboratories are not routinely observing *Cyclospora*. Therefore, awareness was raised among clinicians and laboratories to check for travel history and to test for *Cyclospora* using appropriate methodologies. Identification of non-travel-associated cases for testing is also very challenging. In fact, of the 78 cases identified in 2017 to date, only seven cases were identified as not travel-associated, and the potential source of infection has not yet been found.

Despite the large number of cases in the UK in 2015 and 2016, it is interesting that only two other European countries have reported a small number of cases (France: nine cases in 2016; Belgium: four cases in 2017) [5]. Data from Mexico indicate that after the US and Canada, several European countries (UK, Spain, Germany and France) are in the top 10 in terms of number of visitors to Mexico [17]. The fact that only a very limited number of cases were reported from other European countries could be explained by under-diagnosis, under-reporting and/or because their citizens stayed in other regions of Mexico that were not affected by *Cyclospora*.

Control measures within the UK in 2015 and 2016 were limited to raising awareness about the outbreak, laboratory diagnosis and steps that travellers can take to reduce the risk of becoming infected. Further investigations in UK travellers (e.g. an analytical study) would be important to aid investigations in Mexico. Intercontinental outbreak investigations, risk assessments and management are challenging but collaboration is essential to identify putative vehicles for infection and interrupt transmission.

As the source of previous outbreaks has not been identified, the risk of cyclosporiasis among travellers to Mexico remains high [5]. However, the risk of a local outbreak in the UK as a consequence of an imported case is low due to the unlikely person-to-person transmission. Although the risk to the UK through importation of contaminated food has been assessed in the past [18], it should be revisited in the light of changing food importation and outbreaks in Germany [13] and Sweden [19]. 

The recurring seasonal increase of cyclosporiasis cases is of public health importance due to the detrimental effect that this infection has on symptomatic cases, both during and following travel. Based on the previous UK cyclosporiasis outbreaks, we recommend other European countries to (i) assess, and increase if necessary, *Cyclospora* laboratory testing and reporting capacity, (ii) increase cyclosporiasis risk awareness among travellers to Mexico, (iii) report cyclosporiasis cases early, and (iv) collaborate in outbreak investigations to identify possible sources and to assess the extent of the outbreak.

## Author’s correction

On request of the author group, two authors, Peter Chiodini and Gauri Godbole, were added to this article post-publication on 23 August 2017. These authors had contributed to identification and confirmation of C*yclospora* from cases during the outbreaks from 2015–17, to decisions about sample testing, interpretation of laboratory findings and discussion of methods for genotyping and progression of whole genome sequencing.
