# Author’s correction for Euro Surveill. 2017;22(32)

**DOI:** 10.2807/1560-7917.ES.2017.22.34.30603

**Published:** 2017-08-24

**Authors:** Eurosurveillance editorial team

**Affiliations:** 1 European Centre for Disease Prevention and Control (ECDC), Stockholm, Sweden

On request of the author group, two authors were added post-publication to the article ‘Cyclosporiasis in travellers returning to the United Kingdom from Mexico in summer 2017: lessons from the recent past to inform the future’, published on 10 August 2017. The additional authors are: Peter Chiodini and Gauri Godbole. These authors had contributed to identification and confirmation of *Cyclospora* from cases during the outbreaks from 2015–17, to decisions about sample testing, interpretation of laboratory findings and discussion of methods for genotyping and progression of whole genome sequencing. The change was made on 23 August 2017. 

